# Long-term disease and patient-reported outcomes of a continuous treat-to-target approach in patients with early rheumatoid arthritis in daily clinical practice

**DOI:** 10.1007/s10067-017-3962-5

**Published:** 2018-02-01

**Authors:** G. A. Versteeg, L. M. M. Steunebrink, H. E. Vonkeman, P. M. ten Klooster, A. E. van der Bijl, M. A. F. J. van de Laar

**Affiliations:** 1 Arthritis Centre Twente, Department of Rheumatology, Medisch Spectrum Twente, P.O. Box 50 000, 7500 KA Enschede, The Netherlands; 2 Department of Psychology, Health & Technology, University of Twente, Enschede, The Netherlands; 3 Department of Rheumatology, Isala Hospital, Zwolle, The Netherlands

**Keywords:** Daily clinical practice, Drug-free sustained remission, Implementation, Rheumatoid arthritis, Treat-to-target

## Abstract

Patients in real life may differ from those in clinical trials. The aim of this study is to report 5-year outcomes of a continuous treat-to-target (T2T) approach in patients with rheumatoid arthritis (RA) in daily clinical practice. In the Dutch RhEumatoid Arthritis Monitoring cohort, all patients with a clinical diagnosis of RA were treated according to a protocolled T2T strategy, aimed at 28-joint Disease Activity Score (DAS28) < 2.6. Outcomes were percentages of patients in distinct levels of disease activity, mean course of DAS28 and prevalence of sustained (drug-free) remission. Also, data on functional disability (Health Assessment Questionnaire) and health-related quality of life (Short-Form 36) were examined. Mean DAS28 improved from 4.93 (95% CI 4.81–5.05) at baseline to 2.49 (95% CI 2.35–2.63) after 12 months and remained stable thereafter. Percentages of patients at 12 months with DAS28 < 2.6 (remission), DAS28 ≥ 2.6 and ≤ 3.2 (low disease activity), DAS28 > 3.2 and ≤ 5.1 (moderate disease activity) and DAS28 > 5.1 (high disease activity) were 63, 16, 18 and 3%, respectively. Sustained remission (DAS28 < 2.6 during ≥ 6 months) was observed at least once in 84% of the patients and drug-free remission (DAS28 < 2.6 during ≥ 6 months after withdrawal of all disease-modifying anti-rheumatic drugs) in 36% of the patients. Functional disability and health-related quality of life significantly improved during the first 24 weeks. Continuous application of T2T in real-life RA patients leads to favourable disease- and patient-related outcomes.

**Electronic supplementary material:**

The online version of this article (10.1007/s10067-017-3962-5) contains supplementary material, which is available to authorized users.

## Introduction

Presently, in patients with newly diagnosed rheumatoid arthritis (RA), achieving remission is a realistic goal [1, 2]. Even sustained and drug-free remission is proven to be feasible [3, 4]. This has been achieved during the last decades by early and efficient use of synthetic and biological disease-modifying anti-rheumatic drugs (DMARDs) and by applying treat-to-target (T2T) approaches. In randomised controlled clinical trials, with strict selection of patients and controlled conditions, T2T aiming at remission or low disease activity results in good clinical outcomes [5–7]. It has also been shown that implementation of T2T, especially when using a protocolled treatment strategy, is more effective than a traditional routine care approach [7–9]. Therefore, T2T is now recommended for patients with early RA by the European League Against Rheumatism (EULAR) and the American College of Rheumatology (ACR) [10–12].

Implementing and retaining T2T in daily clinical practice may, however, be a challenge for rheumatologists as well as their patients. Barriers that are frequently mentioned are the complex medication schedules and fear of side effects, doubts about the reliability and validity of composite measurements of disease activity, the applicability of a target of low disease activity for some patients and restrictions in time and resources as well as other logistical aspects [13, 14]. However, in previous publications on the Dutch RhEumatoid Arthritis Monitoring (DREAM) remission induction cohort, successful implementation of T2T in daily clinical practice was demonstrated. Achieving remission within the first year of treatment was shown to be a realistic goal for an important proportion of patients. [2] Furthermore, in most patients, remission was sustained for 6 months or longer during an initial follow-up of 3 years. In addition, patients suffered only limited radiographic damage over this period of time, while physical capacity and health-related quality of life significantly improved [15], illustrating that not only the disease but also the patient benefits from the T2T approach. Adherence to the T2T recommendations was high, which comprised regular assessment of disease activity and protocolled treatment adjustments regarding subsequent disease activity-driven therapeutic steps [16].

Data on long-term results of continuous application of T2T in daily practice are scarce. While many observational studies have evaluated the long-term outcomes of RA, these originate mainly from before the introduction of targeted therapy and the availability of biologicals [1]. Long-term data from more recent randomised controlled clinical trials, using a T2T approach and biologicals, have shown good clinical outcomes [17–20]. However, the generalisability of these results is hampered by the selection of specific patient groups in clinical trials and strict exclusion criteria. Patients seen in real-life practice may differ substantially from those in randomised clinical trials [21]. In this observational study, we therefore describe the 5-year disease and patient-related outcomes of continuous application of a T2T strategy in patients with early RA in daily clinical practice.

## Patients and methods

Between January 2006 and March 2012, all newly diagnosed RA patients at two rheumatology clinics in The Netherlands (Medisch Spectrum Twente Enschede Hospital and Isala Zwolle Hospital) were invited to participate in the DREAM remission induction cohort. Adult patients with a clinical diagnosis of RA (made by an experienced rheumatologist) were included if they had a symptom duration (defined as the time from the first reported symptom to the diagnosis of RA) ≤ 1 year, had a Disease Activity Score in 28 joints (DAS28), calculated using the erythrocyte sedimentation rate (ESR) ≥ 2.6 [22] and had not previously received DMARDs and/or prednisolone. The study was approved by the Medical Ethics Committees of both hospitals (Dutch trial register NTR578). Patients were fully informed and informed consent was obtained from all patients.

### Treatment

Patients were treated according to a protocolled T2Tstrategy aiming at remission, defined as DAS28 < 2.6. The strategy was in line with daily clinical practice and current guidelines. Also, the strategy complied with the Dutch reimbursement regulations regarding prescription of tumour necrosis factor inhibitors (TNFi). At baseline, all patients started methotrexate (MTX) monotherapy at an initial dosage of 15 mg/week which in week 8 could be increased to a maximum dosage of 25 mg/week. Folic acid was taken at the second day after MTX. In case of persistent disease activity in week 12, sulfasalazine (SSZ) was added, starting at a dosage of 2000 mg/day and if necessary increased to a maximum dosage of 3000 mg/day in week 20. TNFi was prescribed at week 24 for patients whose DAS28 remained ≥ 3.2. When the target of DAS28 < 2.6 was reached, medication was left unchanged. In the case of sustained remission (DAS28 < 2.6 for ≥ 6 months), medication was gradually tapered and eventually discontinued. In the case of a disease flare (DAS28 ≥ 2.6), the last effective medication or medication dose was restarted and treatment could subsequently be intensified if necessary. Protocol deviations were allowed in individual patients with contraindications for specific medications. Concomitant treatment with non-steroidal anti-inflammatory drugs, prednisolone at a dosage of ≤10 mg/day and intra-articular corticosteroid injections was permitted. Further details of the study protocol have previously been reported [2].

### Assessments

Patients were assessed at the time of study entry and at every follow-up visit at weeks 8, 12, 20, 24 and every 3 months thereafter. At each assessment, various clinical and patient-reported outcome measures were collected, including the DAS28 (consisting of a 28 tender joint count (TJC28), 28 swollen joint count (SJC28), ESR and patient rating for general health on a 100-mm visual analogue scale (VAS; 0 = best and 100 = worst)). Further, patient-reported outcomes included the disability index of the Dutch version of the Health Assessment Questionnaire (HAQ; ranging from 0 to 3, with high scores indicating more disability) [23] and the component summary scores for physical (PCS) and mental health (MCS) on the 36-item Short-Form Health Survey (SF-36; ranging from 0 to 100, with high scores indicating better health) [24]. DAS28 assessments were performed by well-trained rheumatology nurses and data collection was facilitated by a web-based monitoring application for the physicians as well as for the patients.

### Study outcomes

The main objective of this analysis was to determine the proportion of patients in DAS28 remission at 12, 24 and 52 weeks and after 3 and 5 years and the proportion of patients in low (2.6 ≤ DAS28 ≤ 3.2), moderate (3.2 < DAS28 ≤ 5.1) and high (DAS28 > 5.1) disease activity at those time moments. Secondary outcomes were (1) the change in mean DAS28 scores and median scores of the HAQ and SF-36 (PCS and MCS) over the first 5 years of follow-up, (2) the percentages of patients who achieve sustained DAS28 remission and (3) the time to achieve—as well as the duration of—the first sustained DAS28 remission. Sustained remission was defined as a DAS28 < 2.6 during ≥ 6 consecutive months and could be classified as drug-free when remission was sustained (≥ 6 consecutive months) after the withdrawal of all anti-rheumatic drugs.

### Statistical analyses

The mean change in DAS28 was analysed with a linear mixed model with time as fixed factor. Post hoc paired *t* tests were performed to detect differences between individual time points and baseline and between subsequent time points. To determine the proportion of patients in sustained and drug-free remission, missing values of the DAS28 were imputed by last observation carried forward and only the patients with 5-year follow-up were analysed. Kaplan-Meier survival analysis was performed to assess the time to achieve first sustained DAS28 remission. Changes in HAQ scores and the norm-based component scales (PCS and MCS) of the SF-36 scores were tested using Wilcoxon signed-rank tests. *P* values less than 0.05 were considered significant. Individual changes of 4.4 and 3.1 points on the norm-based component scales of physical health (PCS) and mental health (MCS) of the SF-36 were considered clinically meaningful. For HAQ scores, this individual minimal clinical important improvement was − 0.19 [25]. All analyses were performed using SPSS, version 20.

## Results

### Population and follow-up

From January 2006 to December 2009, a total of 229 patients were included in the cohort. The baseline characteristics of all patients are presented in Table 1. Symptom duration before diagnosis was short, representing a population with very early RA. All patients had active disease at baseline and almost half had at least one radiographic joint erosion. Using the current EULAR definition of erosive disease (at least three erosions in at least three separate joint at specified sites) [26], 16.4% of the patients met this criterion at baseline.

**Table 1:** Baseline characteristics of the patients (*N* = 229)^a^

Characteristic	
Female *n* (%)	145 (63.3)
Age mean ± SD, years	57.5 ± 15.0
BMI mean (± SD), kg/m^2^	26.4 ± 4.6 *N = 220*
Symptom duration median (IQR), weeks	13.0 (8.0–26.0) *N = 228*
RF positive *n* (%)	140/228 (61.4)
Anti-CCP positive *n*/total (%)	129/220 (58.6)
Fulfilment of revised ACR 1987 criteria *n*/total (%)	178/225 (79.0)
Erosive disease (EULAR definition) *n*/total (%)	36/219 (16.4)
Radiographic joint erosion ≥ 1 *n* (%)	102 (46.6)
DAS28-ESR mean (± SD)	4.9 ± 1.1
Number of tender joints (28 assessed) median (IQR)	5 (2–10)
Number of swollen joints (28 assessed) median (IQR)	8 (4–12)
ESR median (IQR), mm/h	28.0 (16.0–42.0)
CRP median (IQR), mg/l	12.0 (5.0–29.3)
Patient’s assessment general health 0–100 VAS median (IQR)	50 (30.0–66.5)
HAQ median (IQR)	1.1 (0.6–1.5) *N* = 191
SF-36 PCS median (IQR)	35.3 (30.0–41.3) *N* = 202
SF-36 MCS median (IQR)	47.5 (39.0–56.3) *N* = 202

*SD* standard deviation, *BMI* body mass index, *IQR* interquartile range, *RF* rheumatoid factor, *anti-CCP* anti-cyclic citrullinated peptide, *ACR* American College of Rheumatology, *EULAR* European League Against Rheumatism, *DAS28-ESR* disease activity score based on 28-joint count calculated using the erythrocyte sedimentation rate, *ESR* erythrocyte sedimentation rate, *CRP* C-reactive protein, *VAS* visual analogue scale, *HAQ* Health Assessment Questionnaire, *SF-36* Short-Form 36 Health Survey, *PCS* physical component summary, *MCS* mental component summary

^a^Values concern the total sample, except indicated otherwise

**Fig. 1:**
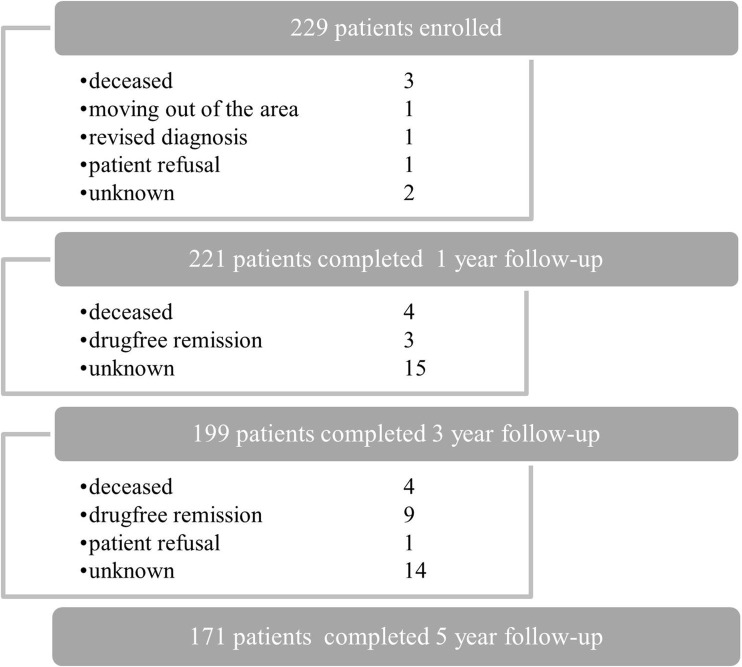
Study sample and dropout

All patients had at least 6 months of follow-up. Data for a follow-up duration of 1, 3 and 5 years were available for 221 (96%), 199 (86.9%) and 171 (74.7%) patients, respectively. Figure 1 shows the reasons for dropout. Patients lost to follow-up were more often men (48.3% (28/58) vs. 32.7% (56/171), *p* = 0.03) of those who continued in the cohort and had shorter symptom duration (median (IQR) 10.0 (6.0–20.0) vs. 15.0 (8.0–26.0), *p* = 0.02). With respect to the other baseline characteristics, patients who were lost to follow-up did not significantly differ from the patients who continued in the cohort.

### Remission rates over time

**Fig. 2:**
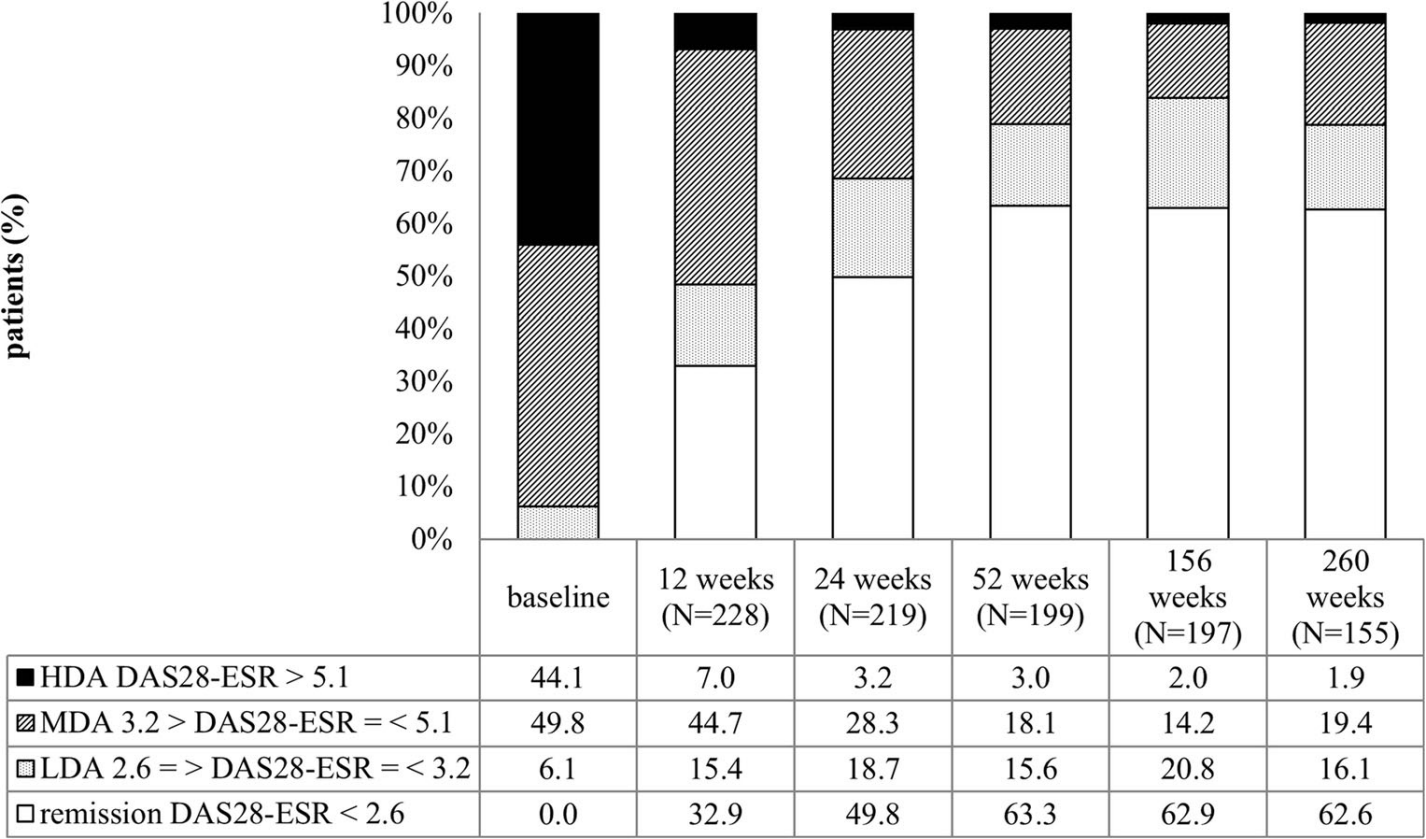
Percentages of patients in different levels of disease activity over the first 5 years of follow-up. DAS-ESR, disease activity in 28 joints, calculated using the erythrocyte sedimentation rate; HDA, high disease activity; MDA, moderate disease activity; LDA, low disease activity

When patients were in sustained remission or low disease activity, they often had fewer visits at their clinics. As a result, we were confronted with missing 3-monthly values during follow-up (25.5%). To determine the numbers of patients in the different levels of disease activity after 3 and 5 years, a window of plus or minus 6 months was used for DAS28 scores. The proportion of patients in DAS28 remission increased in the first year to 63.3% (126/199), and this percentage remained stable thereafter (Fig. 2). After 24 weeks less than 5% (7/219) of the patients was in high disease activity.

### Mean disease activity over time

**Fig. 3:**
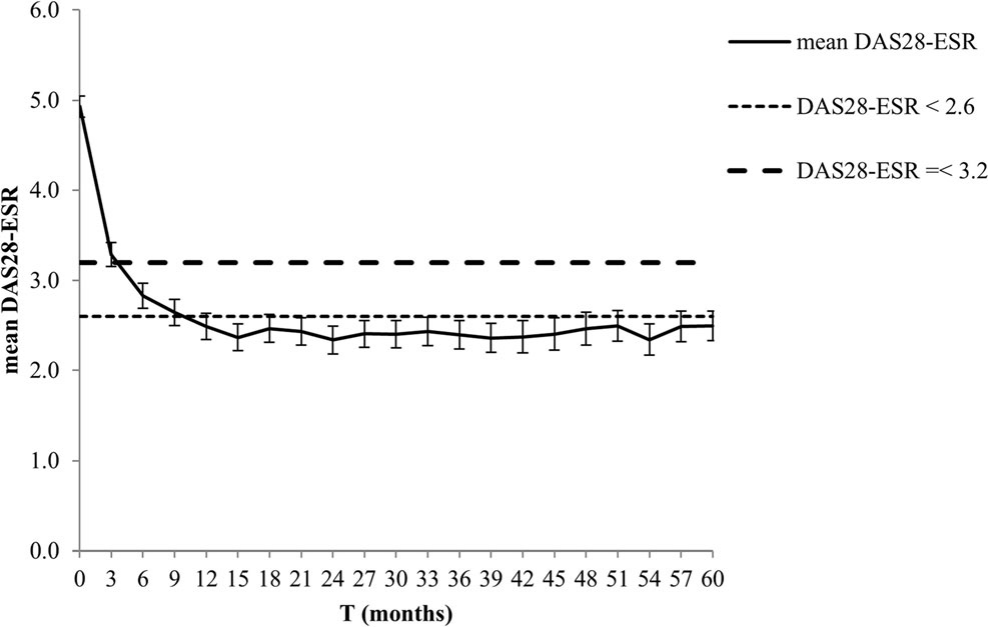
The mean of the disease activity over time. DAS28-ESR, disease activity score in 28 joints, using the erythrocyte sedimentation rate

The mean DAS28 score improved from 4.93 (95% CI 4.81 to 5.05) at baseline to 2.49 (95% CI 2.33 to 2.66) after 5 years (*p* < 0.0001). In the first year, disease activity significantly decreased between each time point with the largest improvement in the first 3 months of treatment. Mean change in DAS28 was − 1.63 (95% CI − 1.80 to − 1.47; *p* < 0.0001) between 3 months and baseline; − 0.47 (95% CI − 0.61 to − 0.33; *p* < 0.0001) between 6 and 3 months; − 0.16 (95% CI − 0.29 to − 0.02; *p* < 0.027) between 9 and 6 months and − 0.14 (95% CI − 0.27 to 0.0; *p* = 0.045) between 12 and 9 months. After the first year of treatment, mean disease activity remained stable below the threshold of DAS28 remission. Figure 3 shows the course of the mean DAS28 score over the first 5 years of follow-up.

### Sustained and drug-free remission

In 84.2% (144/171) of the patients with a follow-up of 5 years, sustained DAS28 remission was observed at least once during the first 5 years of treatment. Of these patients, DAS28 remission was sustained for ≥ 12 months in 79.9% (115/144). The Kaplan–Meier estimate of the median time to the achievement of the first sustained remission was 50 weeks (IQR 37.5–62.5). The median (IQR) duration of the first sustained remission was 97 weeks (52.0–176.0). Of the patients with complete follow-up (*N* = 171) plus the patients who were lost to follow-up because of drug-free remission (*N* = 12), 36.1% (66/183) could stop medication while still maintaining remission for at least 6 months (drug-free remission). Of these 40.9% (27/66) subsequently experienced a disease flare (DAS28 ≥ 2.6) after which 40.7% (11/27) restarted their medication. A further six patients resumed medication without suffering a DAS28 disease flare.

### Medication

During the total follow-up period of 5 years, 38 (16.6%) patients were treated with biologicals, mainly TNF-α inhibitors (adalimumab 60.0%, etanercept 21.7% and infliximab 6.7%). Other biologicals used were abatacept (5.0%), tocilizumab (5.0%) and golimumab (1.7%). Median (IQR) time from baseline to start of the first biological was 53.5 (34.3–119.5) weeks. This first biological was continuously used for a median (IQR) time of 29 (14–72) weeks. About one third of the patients who started a biological (11/38) switched to a second biological after a median (IQR) duration of 41 (31–133) weeks on the first biological. Two thirds (25/38) of the patients did not need a second biological throughout the follow-up period. Nine out of the 11 biological switchers switched only once. One patient was prescribed four and another one was prescribed five different biologicals. In all patients who needed biologicals, the median (IQR) total time of biological use (sum of separate periods) during the follow-up of 5 years was 101 (36–181) weeks. Of them, 25 (65.8%) reached at least one period of sustained remission. In the total sustained remission group (*N* = 144), the medication used to achieve this first sustained remission was as follows: 48.1% (75/156) of the patients were treated with DMARD monotherapy (nearly all on MTX) of which 14.0% were in combination with corticosteroids, 41.0% (64/156) of the patients were prescribed DMARD combination therapy (mainly MTX and SSZ) and 10.3% (16/156) of the patients who achieve sustained remission needed a biological.

### Functional disability

**Fig. 4:**
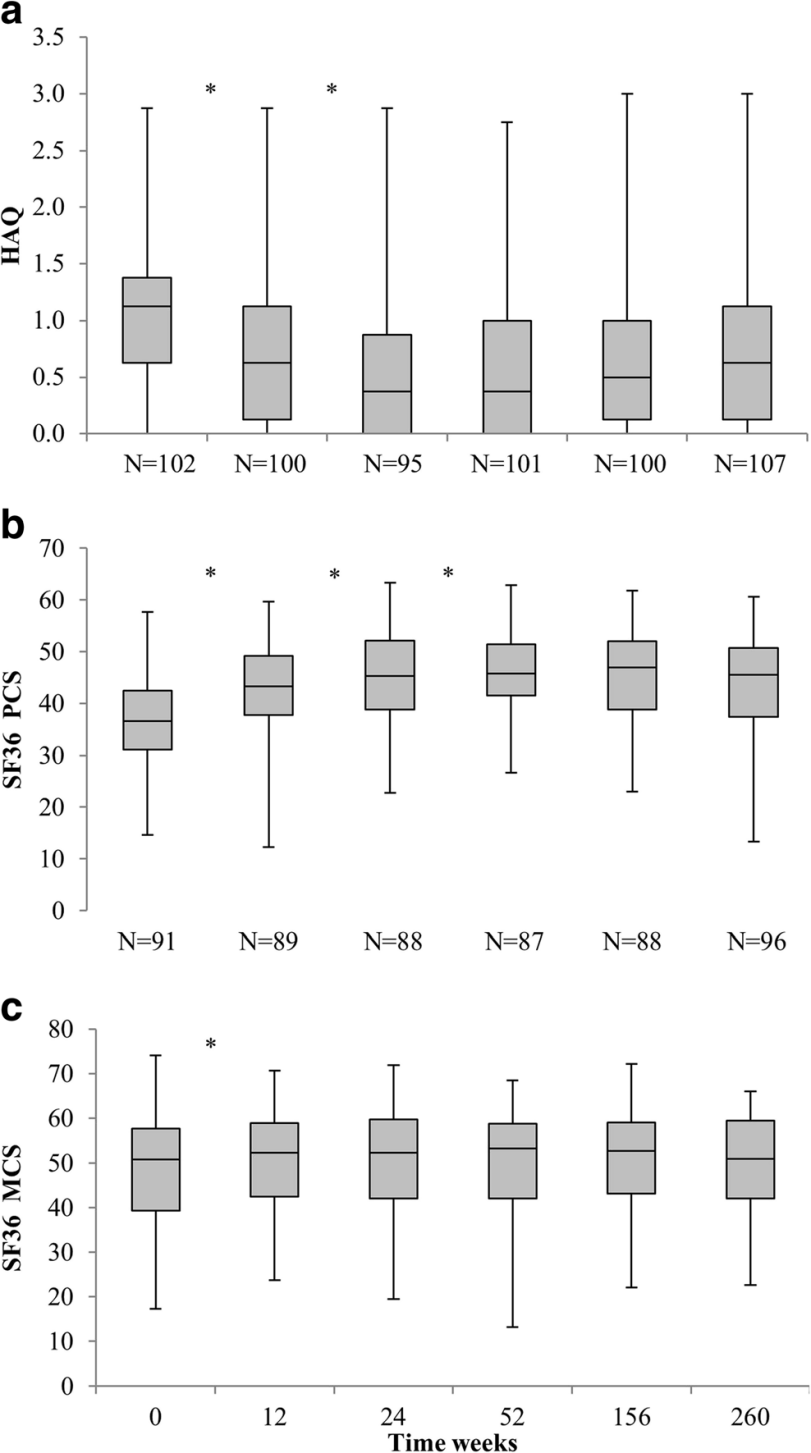
Box plots of Health Assessment Questionnaire (HAQ) and Short-Form 36 Health Survey (SF-36) over 5 years of follow-up. **a** HAQ score, **b** SF-36 physical component summary (PCS) score and **c** SF-36 mental component summary (MCS) score over 5 years of follow-up. *p < 0.05

At baseline, after 12 and 24 weeks and after 1, 3 and 5 years, HAQ scores were available for 201, 196, 178, 183, 155 and 107 patients, respectively. For this outcome, only patients with available HAQ scores at 5 years were analysed. Figure 4a presents the course of the HAQ scores over the first 5 years of follow-up. Median (IQR) HAQ score decreased from 1.125 (0.625–1.375) at baseline to 0.375 (0.000–0.875) after 24 weeks (*p* < 0.001). Thereafter, HAQ scores remained stable. Although median HAQ scores tended to slightly increase between 3 and 5 years, this change (0.125 points) was not significant (*p* = 0.061). Change in individual HAQ score between baseline and 24 weeks was clinically meaningful in 69.6% (112/161) of the patients.

### Health-related quality of life

Baseline, 1-, 3- and 5-year data on quality of life, as measured using the SF-36, were available for 205, 181, 150 and 96 patients. Figure 4b and c presents the box plots of the SF-36 PCS and MCS scores during the first 5 years of follow-up. Both scores significantly improved in the first 3 months of treatment. Median PCS score increased from 36.52 (31.15–42.43) at baseline to 45.71 (41.51–51.42) after 1 year (*p* < 0.001). Median MCS score increased during the first 3 months from 50.81 (39.27–57.67) at baseline to 52.39 (42.51–58.90) after 12 weeks (*p* = 0.031) and remained stable thereafter. Change in individual PCS score between baseline and 1 year was clinically meaningful in 67.7% (113/167) of the patients with available data. In this same time interval, change in individual MCS score obtained clinical relevance in 42.5% (71/167) of the patients with available data.

## Discussion

This study confirms the favourable long-term outcomes of a continuous treat-to-target approach in RA patients in daily clinical practice. Mean disease activity decreased quickly and remained low for the remainder of the follow-up period. The majority of patients reached at least one period of sustained remission in most cases while on conventional synthetic DMARDs. Also, a substantial number of patients achieved drug-free remission. Similarly, functional disability decreased and physical health-related quality of life improved and remained so. The overall use of biological DMARDs was low.

Observational studies in early RA from 1996 to 2007, with a follow-up of 5–10 years, have shown endpoint DAS remission percentages of 23–30% [1]. One of these studies contained a population comparable to our DREAM cohort with regard to baseline disease activity and autoantibody status. This study, in which treatment consisted mainly of methotrexate and sulfasalazine without the application of a T2T principle, demonstrated DAS remission rates of 25 and 20% after 3 and 5 years, respectively [27]. In comparison with these results, our data from the DREAM registry illustrates the major improvement in the management of RA in daily clinical practice that has been realised over the last decades. This improvement has most likely been realised by applying the T2T approach and therefore the efficient use of conventional and biological DMARDs.

There have been several randomised controlled clinical trials, which have proven the effectiveness of T2T strategies. In the CAMERA study, MTX-based tight control during the first 2 years was compared with MTX-based conventional treatment. After 5 years of follow-up, the mean DAS28 decreased from 5.60 to 2.68 and from 5.60 to 2.75 for both treatment arms, respectively [19]. In the BeST study, there were no differences in remission rates after 5 years of follow-up between MTX-based sequential monotherapy, step-up combination therapy, initial combination therapy with prednisone or initial combination therapy with MTX and tumour necrosis factor alpha inhibitors [20]. The total combined DAS remission rate after 5 years was 48% in the BeST study. The difference between this remission rate and the results of our DREAM cohort might be explained by the higher disease activity at baseline, the more stringent inclusion and exclusion criteria and the target of low disease activity in the BeST study.

To date, there is no consensus on how to define sustained remission or drug-free remission, which makes comparison of our results with existing data difficult. In a large Canadian prospective early RA cohort (*N* = 1840) with a follow-up of 5 years in which patients were treated to the discretion of the treating rheumatologist (45.8% MTX combination therapy and 31.7% MTX monotherapy), sustained remission (the more stringent Boolean clinical practice definition; tender joint count ≤ 1 using 28 joints, swollen joint count ≤ 1 using 28 joints and patient global assessment ≤ 1 on a 0–10 scale) for ≥ 6 months was achieved in 25% of the patients [28]. Long-term 10-year follow-up data from the observational Leiden Early Arthritis Cohort and the British Early Rheumatoid Arthritis Study (both started before the use of biologicals and without the T2T principle) showed that sustained DMARD-free remission (the sustained absence of synovitis for at least 1 year after discontinuation of therapy with DMARDs) occurred in 9–15% of RA patients [29]. In the BeST study, after 5 years, 23% of patients achieved drug-free remission (every patient who was able to stop medication regardless of the duration of remission thereafter) with no significant differences between the treatment arms [30].

Our study describes long-term outcome of implementation and continuous application of T2T to RA patients in daily clinical practice. The outcomes are similar to or even better than the results of T2T randomised clinical trials, in which strict selection of patients and controlled conditions were followed. These ‘real-life data’ are of important additional value in the evidence for the effectiveness of a T2T approach in RA patients [31]. The strengths of our study lie in its setting and design. Patients were treated according to current guidelines, and the strategy complied with (Dutch) reimbursement regulations regarding prescription of TNFi. Patients with comorbidities and contraindications for medication did not have to be excluded because deviations from the protocol were allowed. Therefore, results are more likely to be generalisable to other RA populations in the same settings.

Any observational study like this has potential limitations in terms of the possibility of missing data and susceptibility to bias and confounding by indication. The first limitation of our study is the proportion of patients which was lost to follow-up and the amount of missing values. One of the reported reasons for loss to follow-up was sustained remission, which might have led to an underestimation of the actual remission rates. In addition, we were confronted with missing DAS28 values, largely due to less frequent visits to the clinic in case of remission or low disease activity. Linear mixed modelling was used for analysis of the DAS28 course because of the advantage of this method in dealing with missing values. For determining the occurrence of sustained remission, imputation was used by last observation carried forward under the assumption that patients will visit the clinic when the disease flares.

Second, the proportion of patients with positive auto-antibodies rheumatoid factor (RF) and anti-cyclic citrullinated peptide (anti-CCP) in our study is lower than in some recent clinical trials [32, 33], which could be interpreted as a limitation or cast doubt on the accuracy of diagnosis. However, the aim of our study was to describe outcomes of up-to-date treatment of early rheumatoid arthritis in real-life practice. For that reason, we included all patients with a clinical diagnosis of RA made by an experienced rheumatologist after careful consideration of clinical symptoms, laboratory findings and imaging closely mirroring daily practice. In most clinical trials, patients which are diagnosed with RA were selected based on fulfilment of the revised 1987 American College for Rheumatology classification criteria (1987 ACR criteria), including rheumatoid factor. As a consequence, this has likely led to a higher proportion of patients with positive auto-antibodies in these trials. In addition, trials that included early RA patients (diagnosis according to the 1987 ACR criteria) presented proportions of positive auto-antibodies close to our data. For example, the COBRA-light study reported 62% anti-CCP positivity and 58% RF positivity [34] and the BeSt study reported 62% anti-CCP positivity and 66% RF positivity [35].

Third, our target of DAS28-based remission criterion has been criticised regarding its ability to reflect a state of true remission [10]. However, the DAS28 is an applicable and widely used instrument to assess disease activity in daily practice and part of most international reimbursement guidelines for biologicals [36]. Furthermore, our data clearly show that this target resulted in favourable outcomes from the medical as well as the patient perspective.

Finally, this study reports only the results of the implementation of initial step-up MTX monotherapy while in the meantime randomised clinical trials have proven initial combination therapy with different regimes of glucocorticoid use to be superior [33, 37]. Forthcoming cohort studies will have to show whether adjustment of the treatment strategy in daily clinical practice will lead to further improvements.

Implementation and retention of a strict T2T approach in patients with newly diagnosed RA in daily clinical practice leads over a follow-up of 5 years to favourable disease and patient-related outcomes.

## Electronic supplementary material

ESM 1 (DOCX 15 kb)
