# Supplementary material for: Long-term disease and patient-reported outcomes of a continuous treat-to-target approach in patients with early rheumatoid arthritis in daily clinical practice
Source: Clin Rheumatol. 2018 Feb 1;37(5):1189–97. doi: 10.1007/s10067-017-3962-5 (PMC5913385; doi:10.1007/s10067-017-3962-5)
Supplement: Supplementary file 1 [file 10067_2017_3962_MOESM1_ESM.docx]

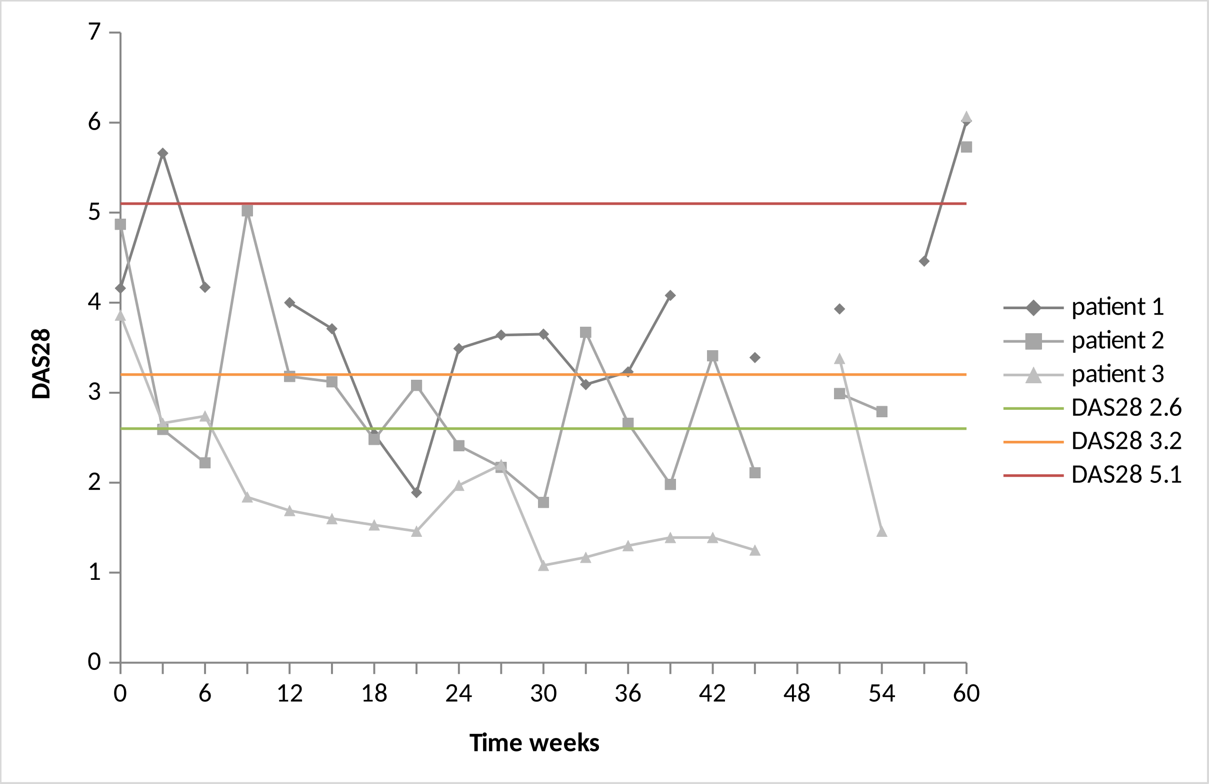

DAS28
Time weeks
patient 1
patient 2
patient 3
DAS28 2.6
DAS28 3.2
DAS28 5.1
